# Timing the Evolutionary Advent of Cyanobacteria and the Later Great Oxidation Event Using Gene Phylogenies of a Sunscreen

**DOI:** 10.1128/mBio.00561-19

**Published:** 2019-05-21

**Authors:** Ferran Garcia-Pichel, Jonathan Lombard, Tanya Soule, Sean Dunaj, Steven H. Wu, Martin F. Wojciechowski

**Affiliations:** a School of Life Sciences, Arizona State University, Tempe, Arizona, USA; b Center for Fundamental and Applied Microbiomics, Biodesign Institute, Arizona State University, Tempe, Arizona, USA; c Department of Cellular and Molecular Biology, Science for Life Laboratory, Uppsala University, Uppsala, Sweden; d Department of Biology, Purdue University, Fort Wayne, Indiana, USA; e Center for Personalized Diagnostic, Biodesign Institute, Arizona State University, Tempe, Arizona, USA; Oregon State University

**Keywords:** UV photobiology, cyanobacteria, deep evolution, secondary metabolism

## Abstract

The biosynthesis of the unique cyanobacterial (oxyphotobacterial) indole-phenolic UVA sunscreen, scytonemin, is coded for in a conserved operon that contains both core metabolic genes and accessory, aromatic amino acid biosynthesis genes dedicated to supplying scytonemin’s precursors. Comparative genomics shows conservation of this operon in many, but not all, cyanobacterial lineages. Phylogenetic analyses of the operon’s aromatic amino acid genes indicate that five of them were recruited into the operon after duplication events of their respective housekeeping cyanobacterial cognates. We combined the fossil record of cyanobacteria and relaxed molecular clock models to obtain multiple estimates of these duplication events, setting a minimum age for the evolutionary advent of scytonemin at 2.1 ± 0.3 billion years. The same analyses were used to estimate the advent of cyanobacteria as a group (and thus the appearance of oxygenic photosynthesis), at 3.6 ± 0.2 billion years before present. *Post hoc* interpretation of 16S rRNA-based Bayesian analyses was consistent with these estimates. Because of physiological constraints on the use of UVA sunscreens in general, and the biochemical constraints of scytonemin in particular, scytonemin’s age must postdate the time when Earth’s atmosphere turned oxic, known as the Great Oxidation Event (GOE). Indeed, our biological estimate is in agreement with independent geochemical estimates for the GOE. The difference between the estimated ages of oxygenic photosynthesis and the GOE indicates the long span (on the order of a billion years) of the era of “oxygen oases,” when oxygen was available locally but not globally.

## Abstract (precis)

The advent of cyanobacteria, with their invention of oxygenic photosynthesis, and the Great Oxidation Event are arguably among the most important events in the evolutionary history of life on Earth. Oxygen is a significant toxicant to all life, but its accumulation in the atmosphere also enabled the successful development and proliferation of many aerobic organisms, especially metazoans. The currently favored dating of the Great Oxidation Event is based on the geochemical rock record. Similarly, the advent of cyanobacteria is also often drawn from the same estimates because in older rocks paleontological evidence is scarce or has been discredited. Efforts to obtain molecular evolutionary alternatives have offered widely divergent estimates. Our analyses provide a novel means to circumvent these limitations and allow us to estimate the large time gap between the two events.

## INTRODUCTION

Most paleoenvironmental models argue that the composition of the Earth’s atmosphere was mildly reducing and anoxic (1–4) before the emergence of oxygenic photosynthesis in the cyanobacteria (5–7). For possibly hundreds of millions of years, the newly produced oxygen reacted in the environment with reduced compounds, so that molecular oxygen was only temporarily and locally available in “oxygen oases” (1, 8, 9) where cyanobacteria were present with sufficient biomass. Once the pool of reduced minerals had been oxidized, though, molecular oxygen started to accumulate in the atmosphere in what is known as the Great Oxidation Event (GOE)—an accumulation that eventually would result in the oxygen-rich atmosphere that we know today (3). Most evidence from the geochemical rock record places the GOE sometime between 2.45 and 2.22 billion years ago (Ga) (2, 4, 10, 11), even though recent estimates show evidence of oxygenated environments as far back as 3 Ga (8, 9).

And yet, the oxygenation of the atmosphere and oceans brought major changes to the ecological nature of the planet, given that oxygen was, and remains, a reactive toxicant to life (12). The GOE also brought about the formation of the stratospheric ozone layer (13), lessening the penetration of highly damaging solar UV radiation in the UVC and UVB ranges (i.e., below 320 nm in wavelength). In contrast, UVA is unaffected by stratospheric ozone absorption, and its harmful photosensitized reactions are mediated by O_2_, so the UVA portion of the solar spectrum (320 to 400 nm) became biologically injurious only after O_2_ started to accumulate in the environment (14, 15). In other words, the accumulation of oxygen in the atmosphere must have turned the UVA into a new ecological stress factor that required novel physiological adaptations. Among such protective adaptations against UVA photodamage, one must count the synthesis of the indole-phenol alkaloid scytonemin, which many cyanobacteria synthesize, excrete, and accumulate in their extracellular sheaths, where it serves as a sunscreen to protect themselves from UVA-mediated photodamage (16). Using sunscreens, however, is a costly photoprotective strategy for microbes, since it requires very large accumulations of pigment to make up for the very short optical path-lengths inherent to microbial cells (17). Sunscreens, by definition, are passive photoprotective mechanisms that require no metabolic activity beyond their initial synthesis. Therefore, microbial sunscreens like scytonemin become of particular fitness value when active photodamage repair mechanisms are impaired or suppressed due to slow metabolic activity or quiescence (18, 19). The distribution of scytonemin producers in nature is consistent with this notion, as they are restricted to habitats where only pulsed growth episodes are possible (terrestrial surfaces, the upper intertidal area, or alpine areas) and no scytonemin producers are found in oceanic or freshwater plankton (14). Scytonemin also undergoes facile reduction even under mildly reducing conditions, losing much of its UV-absorbing capacity and interfering with photosynthetically active radiation (16), no longer providing the photoprotection of the oxidized form. In summary, the evolution of scytonemin makes sense under conditions in which quiescent cyanobacteria were exposed to UVA and oxygen concurrently. In order to maintain oxygenation and photoprotection during periods of organismal quiescence, the oxygenation of the environment must be preserved even when the organisms are not active, so it cannot rely solely on oxygen released by photosynthesis. Yet, since oxygenic photosynthesis is halted while the organisms are inactive, photoprotection provided by scytonemin would only have had a fitness value after the GOE itself. Scytonemin’s advent must thus postdate the GOE.

In this work, we applied divergence-time estimation using relaxed molecular clock models to analyze scytonemin’s biosynthesis genes in an attempt to provide a geochemically independent, minimal age estimate of the GOE. We analyzed the presence, conservation, and synteny of the genes present in the scytonemin operon; studied their phylogenetic relationships; and determined their origin within the broader bacterial evolutionary history. Informative gene phylogenies were then dated using relaxed model analyses that had been calibrated using data from the fossil record. As a result, we obtained multiple, independent evaluations for the timing of relevant events in cyanobacterial evolution. Our results refine previous molecular estimates, are congruent with the accepted geochemical dating, and, importantly, provide a duration estimate of the era of oxygen oases.

## RESULTS

### Scytonemin operon architecture and gene synteny across cyanobacteria.

**FIG 1:**
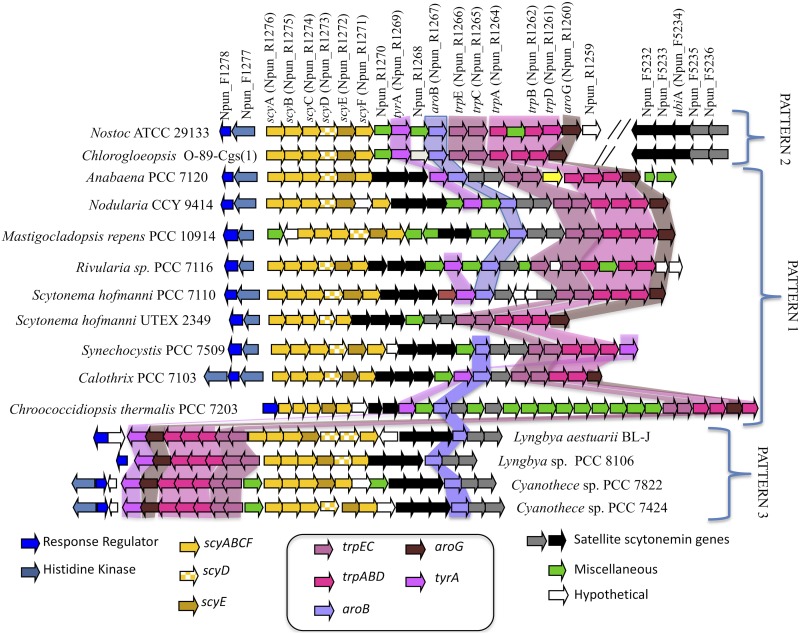
Genomic organization of the cyanobacterial scytonemin operon. Gene nomenclature is based on the annotation of Nostoc punctiforme ATCC 29133. Core biosynthetic genes are shown in yellow. Regulatory genes are shown in blue. Genes shown in black or gray involve a region dedicated to monomer export, and genes shown in various shades of purple/pink code for dedicated aromatic amino acid biosynthetic (AAAB) genes that supply biosynthetic precursors.

The basic comparative structure of the scytonemin operon has been presented elsewhere (20, 21). Here (Fig. 1), we have updated our previous analyses using genomic information currently available in public databases. The core of the operon is made up of the biosynthetic genes *scyABCE*, which are required for biosynthesis of scytonemin from intermediaries of the aromatic amino acid pathway, and the biosynthetic adjuvant *scyF* (22). *scyD*, which is not required for scytonemin production and possesses the characteristics of a pseudogene (22), is missing in at least two strains used in our analyses. This core is otherwise conserved in sequence and shares synteny throughout the phylum. A distinct second region codes for enzymes supplying building blocks to the scytonemin biosynthetic pathway (denoted by various shades of purple in Fig. 1). We refer to those as aromatic amino acid biosynthesis (AAAB) genes. We can divide the AAAB genes into three functional classes: those homologous to tryptophan biosynthesis genes (*trpCE*, *trpABD*); one homolog of a phenylalanine biosynthesis gene, *tyrA*; and *aroBG*, which are homologous to key enzymes in the shikimic acid pathway that feeds precursors to the aromatic amino acid pathways. These AAAB genes represent additional copies of their respective housekeeping versions in all strains that have a scytonemin operon. They are dedicated to scytonemin biosynthesis and coexpressed with the core genes when induced by UVA exposure (23). Scytonemin-operon AAAB genes are highly conserved and frequently share their synteny, particularly for the sequence *trpECABD*-*aroG*. The placement of the *tyrA* and *aroB* genes within the operon seems to be less constrained throughout the phylum. A third group of five highly conserved genes (black/gray in Fig. 1) occurs just downstream of the biosynthetic core in most strains but in a different locus in the genomes of Nostoc punctiforme and *Chlorogloeopsis* sp. (hence the name “satellite genes”). Aside from their distant placement in those two strains, conservation and internal shared synteny of these genes are quite conspicuous. They seem to be involved in the secretion of scytonemin monomers to the periplasm for final condensation (24). A recent contribution (25) has identified the group of satellite genes as the cyanobacterial version of a much more widespread and ancient operon, the “*ebo* operon” conserved among several bacterial phyla and eustigmatophyte plastid genomes. Finally, a set of conserved, homologous regulatory elements, typically forming a two-component regulator, occurs in close proximity to the scytonemin operon, and their presence is also required to allow the synthesis of scytonemin in response to UVA radiation (26).

Three organizational patterns exist among the cyanobacterial genomes with complete scytonemin operons (Fig. 1). The most frequent (pattern 1) consists of the region sequence regulatory/core/satellite/AAAB, with all but the regulatory elements transcribed in the same direction. Pattern 2 (present in Nostoc punctiforme and *Chlorogloeopsis* sp.) is characterized by a transposition of the “satellite” genes onto a distant locus (regulator/core/AAAB/—/satellite). Pattern 3 is found only in nonheterocystous filamentous forms and unicellular strains (*Lyngbya* sp., *Cyanothece* sp.) with the sequence regulators/AAAB/core/satellite. Here, transcription proceeds in opposite directions from the AAAB/core boundary. Since pattern 3 occurs in two lineages that, according to standard cyanobacterial phylogenies (27, 28), diverged earlier from the other cyanobacterial groups that contain these operons, the most parsimonious hypothesis is to assume that this order was the first one to emerge. Then, a rearrangement would have led to pattern 1 before the origin of heterocystous cyanobacteria, and a second, rather recent, rearrangement would have yielded pattern 2 within heterocystous *Nostoc* and close allies. The alternative sequence of pattern 1 first giving rise to pattern 3 and later to pattern 2, however, is also plausible.

In addition to these cyanobacterial genomes with full operons, a few strains also contain evidence of genomic remnants of the scytonemin operon. For example, there are recognizable stretches of the core genes in *Microchaete* strain PCC 7126 and stretches of the AAAB gene region (*trp/aro* clusters, often sharing synteny) in Chamaesiphon minutus PCC 6605, *Fischerella muscicola* PCC 73103, and *Chlorogloeopsis fritschii* PCC 6912. Also, the genomes of *Scytonema hoffmanii* PCC 7110 and *Rivularia* PCC 7116 contain a duplicated version of the AAAB gene region, besides the AAAB locus characteristic of the scytonemin operon.

### Phylogeny of core biosynthetic genes.

The single-gene phylogenies derived from analyses of the core biosynthetic genes *scyABCEF* show clear monophyletic relationships in all strains containing scytonemin operons (see Fig. S1 in the supplemental material), where *scyD* seems to be a nonfunctional paralogue of *scyE* (22). These genes have only low similarity to functionally unrelated genes from other cyanobacteria, bacteria, or fungi (depending on the gene; data not shown). Despite the small number of cyanobacterial genomes that contain the scytonemin operon, *scyABC* phylogenies are congruent with the generally accepted cyanobacterial phylogeny (27, 29) in that the heterocystous groups evolved secondarily, and they lend support to the ancient nature of genomic pattern 3 (see above). For *scyE* (20) and *scyF*, such a pattern is not resolved. In any event, in our data set no cyanobacterial version of any of these genes exists that is not integrated in the *scy* operon, which precludes the placing of their origins in a larger context, so we did not pursue those analyses further.

10.1128/mBio.00561-19.2 FIG S1 Phylogenetic relationships of cyanobacteria derived from neighbor-joining analysis of amino acid sequences of the core scytonemin genes *scyABCF*. Numbers next to nodes represent nonparametric bootstrap support values based on 1,000 replicates. Scytonemin-operon homolog sequences are demarcated by the orange bar, and most closely related genes are demarcated from other bacteria by the blue bar. Download FIG S1, PDF file, 0.3 MB. Copyright © 2019 Garcia-Pichel et al. 2019 Garcia-Pichel et al. This content is distributed under the terms of the Creative Commons Attribution 4.0 International license.

### AAAB gene product phylogenies.

**FIG 2:**
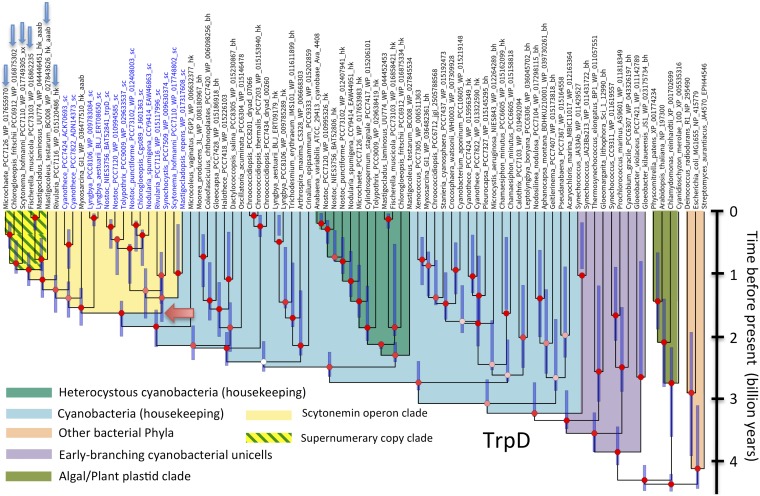
Phylogeny derived from BEAST analyses of TrpD amino acid sequences supports an origin of a scytonemin-associated clade in a single internal duplication of housekeeping genes, shown as exemplary of the two phylogenies (TrpB and TrpC) supporting such an origin. The corresponding phylogeny for TrpC is in Fig. S8. Entries in blue type correspond to homologs found within full scytonemin operons. Those marked by a blue arrow were found in remnant scytonemin operons or correspond to supernumerary homologs. Bayesian posterior probabilities (BPP) at the nodes are color coded as follows: red for BPP ≥ 0.8, pink for 0.8 ≥ BPP ≥ 0.5, and white for BPP ≤ 0.5. The red arrow marks the node corresponding to the most recent common ancestor of the oldest clade containing scytonemin-associated homologs.

**FIG 3:**
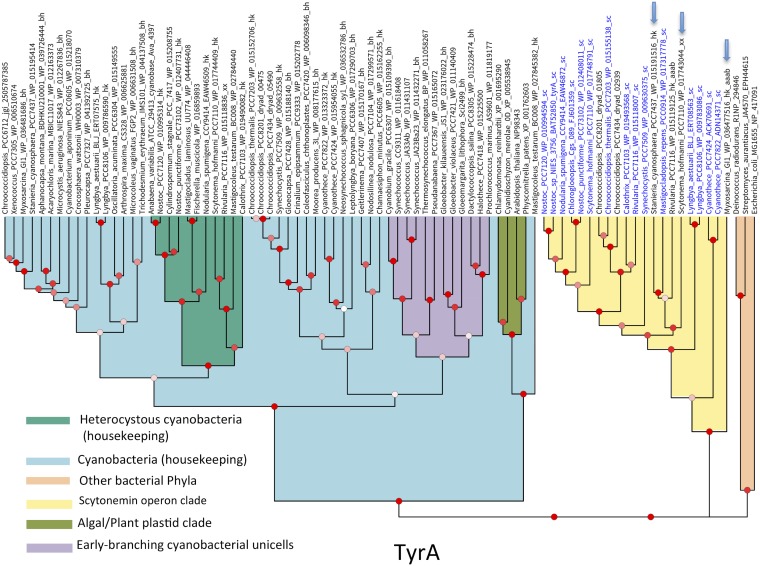
Phylogeny derived from BEAST analyses of TyrA amino acid sequences supports an origin by lateral transfer from bacterial phyla other than cyanobacteria of the homologs in the scytonemin operon. It is shown as exemplary of the three phylogenies (AAAB loci *trpE*, *aroB*, and *tyrA*) supporting such an origin. Phylogenies for the other genes are found in Fig. S5 and S9. Entries in blue type correspond to homologs found within full scytonemin operons. Those marked by a blue arrow were found in remnant scytonemin operons or correspond to supernumerary homologs. Bayesian posterior probabilities (BPP) at the nodes are color coded as follows: red for BPP ≥ 0.8, pink for 0.8 ≥ BPP ≥ 0.5, and white for BPP ≤ 0.5.

**FIG 4:**
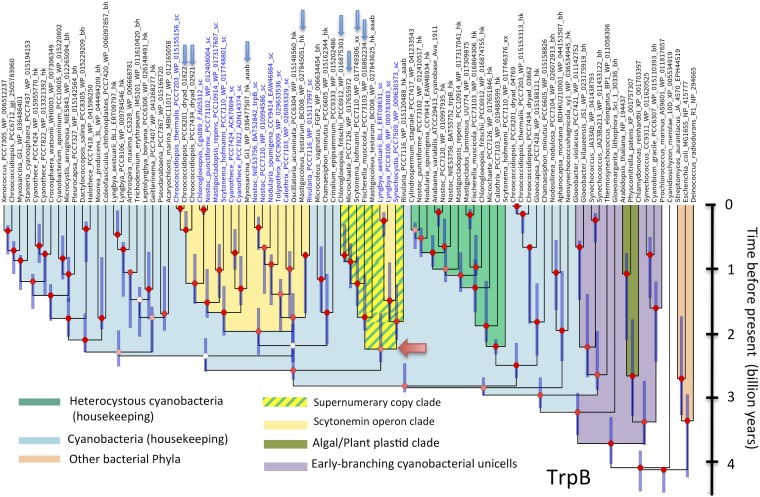
Phylogeny derived from BEAST analyses of TrpB amino acid sequences supports an origin of scytonemin-associated homologs by more than one internal duplication of housekeeping genes, shown as exemplary of the three phylogenies (*trpB*, *trpA*, and *aroG*) supporting such an origin. Phylogenies for the other genes are found in Fig. S6 and S7). Entries in blue type correspond to homologs found within full scytonemin operons. Those marked by a blue arrow were found in remnant scytonemin operons or correspond to supernumerary homologs. Bayesian posterior probabilities (BPP) at the nodes are color coded as follows: red for BPP ≥ 0.8, pink for 0.8 ≥ BPP ≥ 0.5, and white for BPP ≤ 0.5. The red arrow marks the node corresponding to the most recent common ancestor of the oldest clade containing scytonemin-associated homologs.

The fact that the scytonemin-dedicated AAAB genes typically represent additional versions of their respective housekeeping cognates (20) suggests that they may have evolved by duplication of these housekeeping genes in ancestral cyanobacteria. This was of much interest because, if confirmed, the timing of these duplications would represent a conservative estimate of the evolutionary origin of scytonemin. To test this hypothesis, we constructed phylogenetic trees from analyses of the amino acid sequences of each AAAB gene, including all bona fide orthologs present in *scy* operons available from cyanobacteria, some of those found in scytonemin operon remnants, and a phylogenetically representative set of housekeeping orthologs from the cyanobacteria and plant plastids, and included some from other bacterial phyla, to serve as outgroups. If this hypothesis were correct, one would expect to find the *scy* operon orthologs sharing an ancestor within their respective cyanobacterial lineages. Using translated amino acid sequences, we constructed preliminary neighbor-joining trees to explore general relationships (Fig. S1 to S4). These trees clearly showed that the different scytonemin-dedicated AAAB proteins tended to cluster together, although not always branching within the main housekeeping group of AAAB enzymes from cyanobacteria, but the trees did not show high support at relevant nodes. The same data sets were then used to obtain full Bayesian trees derived from BEAST analyses (Fig. 2 to 4; also Fig. S5 to S9). Our neighbor-joining and Bayesian results were largely congruent, and they have been summarized in Table 1. For five of the eight loci, the hypothesis of an internal duplication of the AAAB genes within their cyanobacterial housekeeping counterparts was well supported (Bayesian posterior probability [BPP] = 1 in AroG, TrpA, TrpC, and TrpD, and BPP > 0.8 in TrpB). For the remaining three (TrpE, AroB, and TyrA) (Fig. 3 and Fig. S5 and S9), the Bayesian phylogenies strongly support an ancient recruitment of these genes into the operon through lateral gene transfer from other bacterial lineages (BPP = 1 in the three cases), while their respective cyanobacterial housekeeping clades also show strong support (BPP ≥ 0.99).

**TABLE 1:** Summary of phylogenetic results for the evolution of aromatic amino acid biosynthesis loci contained in the scytonemin operon of cyanobacteria as determined from model-based Bayesian analyses of relevant locus-specific data sets

Locus	Whether orthologs form monophyletic group	Whether scytonemin clade(s)^*a*^ originate(s) within cyanobacterial phylogeny	Housekeeping phylogeny consistent with cyanobacterial phylogeny^*b*^
*trpB*	Unclear	Yes	Yes
*trpC*	Yes	Yes	Yes
*trpD*	Yes	Yes	Yes
*trpA*	No, two clades	Yes	Yes
*aroG*	No, multiple clades	Yes	Yes
*trpE*	Yes	No, lateral transfer	Yes
*aroB*	Yes	No, lateral transfer	Yes^*c*^
*tyrA*	Yes	No, lateral transfer	Yes

a Including a clade of supernumerary and scytonemin operon remnant copies.

b See text for criteria.

c However, it contains two clades of heterocystous cyanobacteria.

10.1128/mBio.00561-19.3 FIG S2 Phylogenetic relationships of cyanobacteria derived from neighbor-joining analysis of amino acid sequences of the *aroB* (114 taxa, 219 characters) and *aroG* (78 taxa, 341 characters) genes. Numbers next to selected nodes represent nonparametric bootstrap support values based on 500 replicates, but only those above 70% are marked. Scytonemin-operon gene homolog sequences are shown in blue type, and supernumerary homologs (beyond housekeeping and scytonemin associated) and those in remnant scytonemin operons are in green type. Download FIG S2, PDF file, 0.1 MB. Copyright © 2019 Garcia-Pichel et al. 2019 Garcia-Pichel et al. This content is distributed under the terms of the Creative Commons Attribution 4.0 International license.

10.1128/mBio.00561-19.4 FIG S3 Phylogenetic relationships of cyanobacteria derived from neighbor-joining analysis of amino acid sequences of the *trpA* (89 taxa, 235 characters), *trpB* (89 taxa, 395 characters), *trpC* (95 taxa, 233 characters), and *trpD* (84 taxa, 294 characters) genes. Numbers next to selected nodes represent nonparametric bootstrap support values based on 500 replicates, but only those above 70% are marked. Scytonemin-operon gene homolog sequences are shown in blue type, and supernumerary homologs (beyond housekeeping and scytonemin associated) and those in remnant scytonemin operons are in green type. Download FIG S3, PDF file, 0.2 MB. Copyright © 2019 Garcia-Pichel et al. 2019 Garcia-Pichel et al. This content is distributed under the terms of the Creative Commons Attribution 4.0 International license.

10.1128/mBio.00561-19.5 FIG S4 Phylogenetic relationships of cyanobacteria derived from neighbor-joining analysis of amino acid sequences of the *trpE* (94 taxa, 354 characters) and *tyrA* (83 taxa, 137 characters) genes. Numbers next to selected nodes represent nonparametric bootstrap support values based on 500 replicates, but only those above 70% are marked. Scytonemin-operon gene homolog sequences are shown in blue type, and supernumerary homologs (beyond housekeeping and scytonemin associated) and those in remnant scytonemin operons are in green type. Download FIG S4, PDF file, 0.1 MB. Copyright © 2019 Garcia-Pichel et al. 2019 Garcia-Pichel et al. This content is distributed under the terms of the Creative Commons Attribution 4.0 International license.

10.1128/mBio.00561-19.6 FIG S5 Phylogeny for scytonemin-dedicated AAAB genes of AroB amino acid sequences derived from BEAST analyses. Entries in blue type correspond to homologs found within full scytonemin operons. Bayesian posterior probabilities (BPP) at the nodes are color coded as follows: red for BPP ≥ 0.8, pink for 0.8 ≥ BPP ≥ 0.5, and white for BPP ≤ 0.5. Download FIG S5, PDF file, 0.6 MB. Copyright © 2019 Garcia-Pichel et al. 2019 Garcia-Pichel et al. This content is distributed under the terms of the Creative Commons Attribution 4.0 International license.

10.1128/mBio.00561-19.10 FIG S9 Phylogeny derived from BEAST analyses of TrpE amino acid sequences for scytonemin-dedicated AAAB genes. Entries in blue type correspond to homologs found within full scytonemin operons. Those marked by a blue arrow were found in remnant scytonemin operons or correspond to supernumerary homologs. Bayesian posterior probabilities (BPP) at the nodes are color coded as follows: red for BPP ≥ 0.8, pink for 0.8 ≥ BPP ≥ 0.5, and white for BPP ≤ 0.5. Download FIG S9, PDF file, 0.5 MB. Copyright © 2019 Garcia-Pichel et al. 2019 Garcia-Pichel et al. This content is distributed under the terms of the Creative Commons Attribution 4.0 International license.

A recurrent pattern in most phylogenies (except for AroB) was that AAAB homologs assignable to supernumerary gene copies (additional to the housekeeping and scytonemin-operon versions, many of them belonging to remnant scytonemin operons) were nested within the clades containing “true” scytonemin-operon copies, indicating that they indeed represented old scytonemin-associated copies or had originated as secondary duplications of scytonemin homologs. Thus, in the following narrative, we include such traceable homologs in our definition of the scytonemin clade for any given AAAB phylogeny. In AroB (Fig. S5), a well-supported clade of supernumerary copies that was internal to the scytonemin clade has been related to the biosynthesis of secondary metabolites other than scytonemin (25).

Among five loci that lent support to an origin within the cyanobacteria, phylogeny reconstructions placed the scytonemin-operon AAAB orthologs in well-supported monophyletic groups in two cases (TrpD, BPP = 0.97; TrpC, BPP = 0.98) (Fig. 2 and Fig. S8). The node marking the most recent common ancestor of the scytonemin operon in these cases is obvious. In the other three genes there was always a main clade of scytonemin sequences, but a few of them branched outside this main group. For instance, in TrpA (Fig. S7), *Lyngbya* homologs were clearly paraphyletic to the main scytonemin-associated clade, indicating the presence of two independent recruitment duplications from the housekeeping clade. Incidentally, *Cyanothece* and *Lyngbya* scytonemin operons are those presenting the ancient organizational “pattern 3” and show the deepest-branching patterns for ScyABC (Fig. S1) in our trees. A similar case is observed in AroG (Fig. S6), where the scytonemin homologs of *Cyanothece* are paraphyletic to the main scytonemin-associated clade. Finally, for TrpB sequences, two scytonemin-operon clades could be defined (0.8 > BPP > 0.5), a main clade containing most homologs and a second clade containing the *Lyngbya* homologs and a group of supernumerary copies (Fig. 4). The poor resolution of that part of the tree, however, implies that one cannot exclude the possibility that the separation in two clades may not represent the true evolutionary history of these genes. In the latter three genes, we take the common ancestors of the most deeply branching clade as oldest evidence for the existence of the scytonemin operon.

10.1128/mBio.00561-19.7 FIG S6 Phylogeny derived from BEAST analyses of AroG amino acid sequences for scytonemin-dedicated AAAB genes. Entries in blue type correspond to homologs found within full scytonemin operons. Those marked by a blue arrow were found in remnant scytonemin operons or correspond to supernumerary homologs. Bayesian posterior probabilities (BPP) at the nodes are color coded as follows: red for BPP ≥ 0.8, pink for 0.8 ≥ BPP ≥ 0.5, and white for BPP ≤ 0.5. The red arrow marks the node corresponding to the most recent common ancestor of the oldest clade containing scytonemin-associated homologs. Download FIG S6, PDF file, 0.5 MB. Copyright © 2019 Garcia-Pichel et al. 2019 Garcia-Pichel et al. This content is distributed under the terms of the Creative Commons Attribution 4.0 International license.

10.1128/mBio.00561-19.8 FIG S7 Phylogeny derived from BEAST analyses of TrpA amino acid sequences for scytonemin-dedicated AAAB genes. Entries in blue type correspond to homologs found within full scytonemin operons. Those marked by a blue arrow correspond to those found in operon remnants operons or to supernumerary copies. Bayesian posterior probabilities (BPP) at the nodes are color coded as follows: red for BPP ≥ 0.8, pink for 0.8 ≥ BPP ≥ 0.5, and white for BPP ≤ 0.5. The red arrow marks the node corresponding to the most recent common ancestor of the oldest clade containing scytonemin-associated homologs. Download FIG S7, PDF file, 0.5 MB. Copyright © 2019 Garcia-Pichel et al. 2019 Garcia-Pichel et al. This content is distributed under the terms of the Creative Commons Attribution 4.0 International license.

10.1128/mBio.00561-19.9 FIG S8 Phylogeny derived from BEAST analyses of TrpC amino acid sequences for scytonemin-dedicated AAAB genes. Entries in blue type correspond to homologs found within full scytonemin operons. Those marked by a blue arrow were found in remnant scytonemin operons or correspond to supernumerary homologs. Bayesian posterior probabilities (BPP) at the nodes are color coded as follows: red for BPP ≥ 0.8, pink for 0.8 ≥ BPP ≥ 0.5, and white for BPP ≤ 0.5. The red arrow marks the node corresponding to the most recent common ancestor of the oldest clade containing scytonemin-associated homologs. Download FIG S8, PDF file, 0.6 MB. Copyright © 2019 Garcia-Pichel et al. 2019 Garcia-Pichel et al. This content is distributed under the terms of the Creative Commons Attribution 4.0 International license.

In order to determine if the AAAB gene phylogenies are congruent with conventional knowledge of cyanobacterial evolution, we tested them for compliance with a set of criteria that can be derived from current molecular phylogenetic and paleontological evidence (5, 30–32). The criteria included the following: (i) the deepest/earliest branches should correspond to unicells (minimally *Gloeobacter*, but often including *Gloeomargarita*, and thermophilic *Synechococcus* homologs), (ii) followed in distance from the root by simple filamentous forms, with (iii) clades of heterocystous forms appearing later (i.e., more derived) within the cyanobacterial radiation. Additionally, (iv) the algal/plant plastid lineages had to be monophyletic and branch off close to the base of the cyanobacterial lineage. All eight AAAB housekeeping protein trees met these criteria if representatives of the groups involved were present (Table 1; Fig. 2 to 4 and Fig. S5 to S9). Two potential, if likely inconsequential, exceptions are to be noted. First, one could have doubts about the compliance of TrpD with additional criterion iv. Second, the marine unicellular *Prochlorococcus* clade in all trees branched deeper/earlier than in standard 16S rRNA gene trees, potentially reflecting long branch artifacts due to the higher divergence in the well-described process of genome simplification that these taxa have experienced (33).

### Timing of evolutionary events.

**FIG 5:**
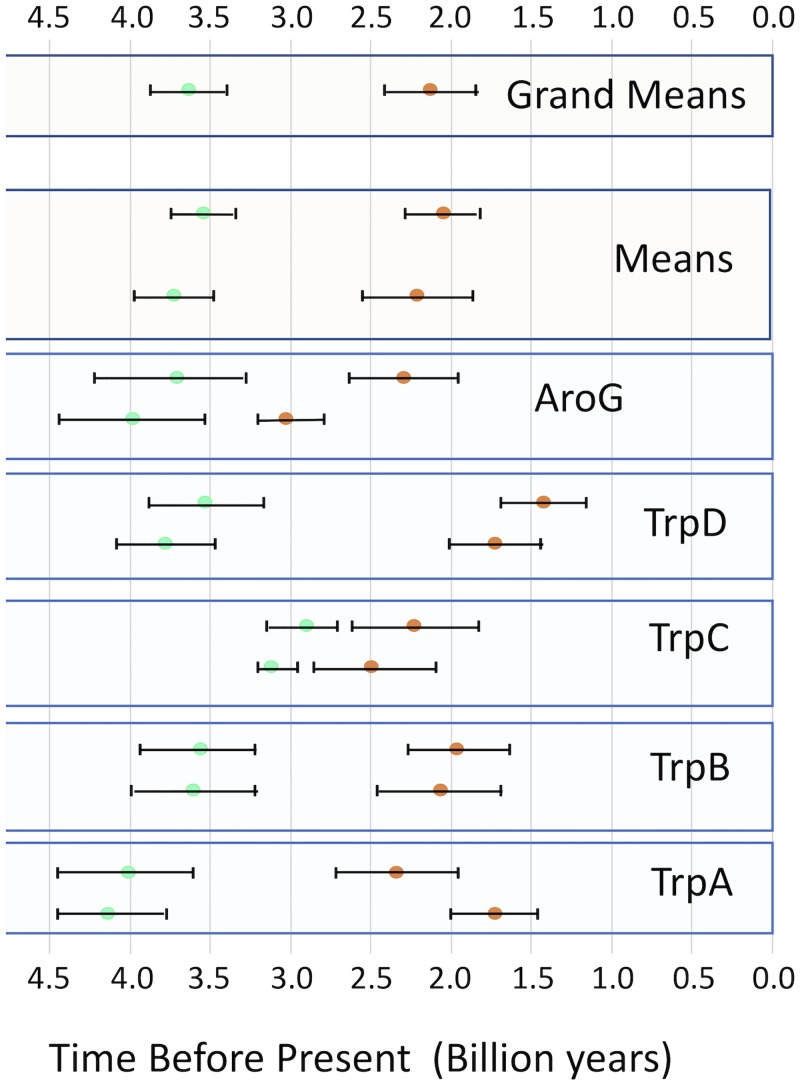
Time estimates for the origin of cyanobacteria (blue-green) and scytonemin (orange) derived from relaxed molecular clock models applied to the phylogeny of gene products associated with the scytonemin operon. Each of the lower 5 boxes corresponds to a gene product as marked. In each of these boxes, the upper estimates used an age of 0.9 billion years for the common ancestor of all heterocystous cyanobacteria, whereas the lower estimates used an age of 2.1 billion years. Error bars are 95% CI for each estimate. The two uppermost boxes include averages of single-gene estimates for each of the two age scenarios (Means) or for all estimates (Grand Means).

To calibrate the timing of the evolutionary appearance of scytonemin and to avoid the use of geochemical markers that rely on redox changes, we used exclusively fossil evidence and refrained from using geochemically derived constraints other than the age of the Earth. We also avoided using dates derived from molecular fossils (hopanoids), as these have recently been discredited (34). We also refrained from using the 3.3-Ga age of the oldest cyanobacterium-like fossils of Schopf and Packer (35), which have been used by others (29, 36), because their biogenicity has been effectively challenged (37). We used an absolute 2-point calibration. For the first point, we chose the appearance of filamentous cyanobacteria at 2.7 Ga based on dating of the Tumbiana microfossils (38), which remains uncontroversial and is backed up by the contemporaneous evidence for structures built exclusively by filamentous cyanobacteria (38) and the presence of oxygen bubbles in fossil stromatolites (39). Given that the deepest branches in our trees are populated by unicellular strains, we assigned this first calibration point to the node containing the oldest filamentous strains. The second point was the age of the clade comprising the orders *Nostocales* and *Stigonematales* (heterocyst forming). An age of 2.1 Ga is widely used for this point (29, 31, 36, 40), but a recent publication has challenged the interpretation of the original fossils (41), claiming that the oldest fossils for this group are from the base of the Tonian (some 0.9 Ga old), at the beginning of the Neoproterozoic. For our interpretation, the new fossils are also problematic in that they are too large to be cyanobacteria and do not show heterocysts. Given this situation, we have pragmatically constrained the origin of the heterocystous clade to each of the two possible dates (2.1 and 0.9 Ga) and have run the model-based analyses independently. The results proved rather insensitive to this source of uncertainty (Fig. 5). The models were only otherwise constrained with the condition that the deepest node in each tree could not be older than 4.5 Ga, the age of the Earth. All AAAB genes but those showing an origin in lateral gene transfer from other phyla were used for the divergence time estimation, and the results are summarized in Fig. 5. The estimates for the minimal age of the scytonemin operon were determined as the age of the common ancestor of all bona fide scytonemin-operon homolog proteins in the trees that had a monophyletic distribution. For the genes that produced paraphyletic scytonemin clades, the estimated age of the earliest-branching clade was used instead. The estimates were obtained as the geometric mean of the probability distribution for the relevant node and ranged from 3.0 to 1.7 Ga, with a grand mean (*n* = 10) of 2.12 (±0.28, 95% CI) Ga. Similarly, we estimated the age of cyanobacteria (=oxyphotobacteria) as the age of the respective nodes encompassing all housekeeping homologs; they ranged from 2.7 to 4.1 Ga, with a mean estimate of 3.63 ± 0.24 Ga.

**FIG 6:**
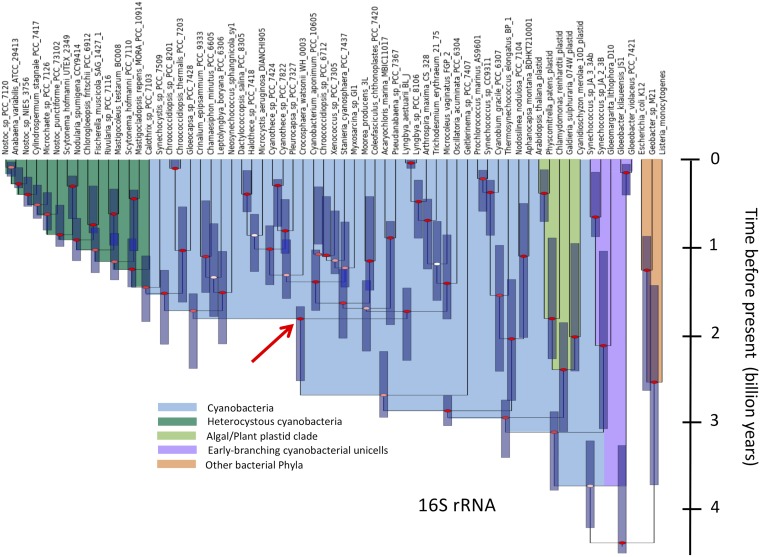
Phylogeny derived from BEAST analyses of full 16S rRNA sequences of cyanobacteria. Bayesian posterior probabilities (BPP) at the nodes are color coded as follows: red for BPP ≥ 0.8, pink for 0.8 ≥ BPP ≥ 0.5, and white for BPP ≤ 0.5. The red arrow marks the node corresponding to the most recent common ancestor of the clades encompassing scytonemin-operon containing genomes.

Once the evolutionary history of the scytonemin operon has been traced through the appropriate AAAB genes, it is possible to interpret phylogenies of standard marker genes under that light. Using the same set of strains as in the AAAB phylogenies, and the same time calibrations, 16S rRNA trees (Fig. 6) yielded topologies congruent with the conventional criteria stated above. The estimates for the origin of scytonemin-operon-containing cyanobacteria (2.1 to 2.6 Ga) and the origin of cyanobacteria (3.7 to 4.1 Ga) here are remarkably consistent with the estimates based on the AAAB genes. Alternatively, a concatenated tree of conserved genes could have been used to decrease the variation in age estimates, and any potential bias, when one attempts to obtain an evolutionary history of a species or group thereof, as long as all genes shared a congruent evolutionary history. It should prove interesting to see if our findings using multiple genes within the operon would find confirmation using concatenated core genes, as they did when using the widely used 16S rRNA gene for this purpose.

## DISCUSSION

The high degree of conservation in sequence, synteny, and regulatory elements of the scytonemin operon speaks for a genetic locus of secondary metabolism that has presumably conferred a fitness advantage to cyanobacteria through much of their history. Although many cyanobacteria possess this locus, they are by no means a majority of those strains with fully sequenced genomes. This likely reflects the well-known preferential distribution of scytonemin among cyanobacteria from environments that allow only periodic growth (16). The operon appears to have originated once but has probably been lost multiple times during diversification, as attested by the presence of operon remnants in some strains. The synteny of these genes has been also quite conserved, although a major reorganization apparently took place early on, from an ancient arrangement now typical of filamentous and secondarily unicellular/colonial cyanobacteria, to yield a more modern one characteristic of the heterocystous groups. The operon itself is conspicuously absent from many of the sequenced isolates in the earliest branches of cyanobacterial diversification and from the early-branching lineage that gave rise to plastids (27), implying that scytonemin appeared sometime early during the diversification of cyanobacteria but not at its root. This is consistent with the notion of a time gap between the appearance of cyanobacteria and the GOE, during which a microbial UVA sunscreen would have provided little fitness value (see the introduction). The presumably most ancient core part of the operon benefited from the recruitment of genes dedicated to supplying the aromatic amino acid precursors. Our analyses clearly suggest that three AAAB genes were acquired through lateral transfer and the rest by duplication of housekeeping genes. The latter allowed us to obtain multiple independent estimates of the sequence of events and the timing of their duplication.

Our estimates of the age of the scytonemin operon should be considered conservative in that an ancient but functional operon must have existed that contained only the core genes before the AAAB genes were recruited into it in order to optimize precursor supply. Estimates for the minimum age of the scytonemin operon, with an average of 2.12 ± 0.28 Ga, are consistent with a variety of previous estimates for the GOE (2, 4, 10, 11). It is a possibility that including the “remnant genes” in the scytonemin clade may have artificially pushed back our estimate of its origin, if the remnants were nonfunctional and thus not subject to purifying selection; unfortunately, we can provide no evidence for their functional status one way or the other. In any event, our estimates have added value in that they do not rely on any geochemical evidence; they are based exclusively on molecular evolution models and fossil evidence and thus constitute a truly independent avenue to the same end. In view of the final estimates, this alternative avenue converges with geochemical estimates.

Our data sets also allow us to obtain multiple estimates of the origin of the cyanobacteria (=oxyphotobacteria), perhaps the single most important evolutionary event in the history of the Earth. While minimum ages for the presence of cyanobacteria can principally be derived from the (micro)fossil record, rocks of this age are exceedingly rare and fossils in them are few and poorly preserved. There is little doubt among paleontologists that the actual origin of cyanobacteria very likely and significantly predates the best-preserved, noncontroversial fossils (7). Using the molecular evolutionary models anchored with fossil calibrations may be a robust way of circumventing this limitation. And yet, the use of fossil markers, particularly the oldest ones, remains a source of uncertainty in this and other studies (29, 31, 36, 40), even when we strived to select a deep-time marker that was congruent among various lines of evidence. Importantly, we also contend that the convergence of the estimates of the age of scytonemin with the already-available geochemical estimates of the GOE provides an internal cross-disciplinary check on the goodness of our methodology, lending additional credence to our concurrent estimates of the age of the most recent common ancestor of cyanobacteria at 3.63 ± 0.24 Ga. Results from similar analyses carried out on 16S rRNA genes (but using the same time markers) from the same group of strains are consistent with this timing. Rigorously, however, the inference, based on the redox chemistry of scytonemin, that its advent must postdate the GOE (see the introduction), may be regarded as too indirect. If this stance is taken, then the current results can be interpreted as meaning that scytonemin evolution indeed postdated the geochemically established GOE, rather than adding an independent estimate to it.

The key innovation that the evolution of oxygenic photosynthesis represents meant that for the first time a phototroph could use a ubiquitous source of electrons, water, for the reduction of CO_2_. This liberated photosynthesis from the ecological servitude of more localized and limited sources of reductant, possibly enabling the colonization of the continents (42, 43), where the occurrence of scytonemin is prevalent (44). From our study, this event can be traced back deep into the Archaean Eon. These estimates are remarkably close to the indirect, synthetic view offered in Schopf’s 2011 review (45). In contrast, however, recent molecular clock estimates using concatenated central metabolism genes (46) provide much more recent dates for the same event, almost coincident with that of the GOE. That study, however, used calibrations based on the plant fossil record applied to the branch of the cyanobacterial phylogeny, even when it can hardly be expected that conserved genes in the free-living cyanobacteria and their symbiotic relatives within plants be subject to similar evolutionary rates.

The absence of concurrent time estimates for the origin of cyanobacteria and the GOE based on the same data sets has prevented rigorously gauging the length of the lag period between the appearance of oxygenic photosynthesis and the GOE, a time of “oxygen oases,” geochemical “whiffs of oxygen” (1), exceedingly extreme photochemical harshness (15), and likely great evolutionary significance. Our estimates, whose means differ by 1.5 billion years, suggests that it may have taken cyanobacteria this long to change the Earth’s Archaean biosphere from neutral/reducing to oxidizing in nature, a period 3-fold as long as the age of the oldest green plants or metazoans. It is conceivable that fully oxygenated niches, some perhaps quite large, existed during this time in closed basins, providing opportunities for the evolution of biological oxidative metabolism and oxidative damage defense mechanisms in multiple, independent lineages (47) and, in time, for the eventual tipping point in the evolutionary trajectory of life that the GOE represents.

## MATERIALS AND METHODS

### Genomic data and reconstructions.

DNA sequences of the genes of the scytonemin operon of Nostoc punctiforme ATCC 29133 (PCC 73102) that had homologs in the aromatic amino acid biosynthesis (AAAB) pathway, as well as the corresponding AAAB amino acid sequences, were used as queries for similarity searches (see Text S1 in the supplemental material). Initial BLASTp searches (48) were carried out against GenBank on a broad selection of genomes from cyanobacteria and a few representatives from bacteria and plastid-bearing eukaryotes (Text S1). Owing to the high number of homologs detected, however, genomes were recovered from GenBank (www.ncbi.nlm.nih.gov/genome/), CyanoBase (http://genome.microbedb.jp/cyanobase/), JGI (https://genome.jgi.doe.gov/), and Dryad (https://datadryad.org/) to build a local cyanobacterial database that was both taxonomically representative of this phylum and enriched in organisms with a scytonemin operon or particular relevance for subsequent calibration purposes (Text S1). The final BLASTp searches on cyanobacteria were carried out with default parameters and permissive E values (<1e−05) against that local database.

10.1128/mBio.00561-19.1 TEXT S1 (A) Genomes used in the analyses. (B) Sequences from Nostoc punctiforme PCC 73102 (ATCC 29133) used as seeds in the BLAST analyses. Download Text S1, PDF file, 0.1 MB. Copyright © 2019 Garcia-Pichel et al. 2019 Garcia-Pichel et al. This content is distributed under the terms of the Creative Commons Attribution 4.0 International license.

For each relevant protein-coding gene detected in a cyanobacterial genome, the five protein-coding sequences up- and downstream of that locus were recorded to determine if they were likely part of a larger scytonemin operon (“SC copies”) or isolated and therefore likely involved in housekeeping (“HK copies”) or other functions. The proteins tentatively annotated as SC copies were further evaluated by examining up- and downstream sequences until there were three proteins in a row that were dissimilar to proteins in the *N. punctiforme* scytonemin operon in each direction. This approach was used to delimit the scytonemin protein cluster in all of the chosen taxa and consequently provide greater confidence in annotating them as SC or HK copies. In some cases, we found supernumerary copies (beyond SC or those initially labeled HK). These were also included, and we determined *post hoc* if they were related to the main HK or the SC lineages. Preliminary alignments of each gene were carried out using MUSCLE v3.8.31 (49), the sequences were trimmed using the program NET of the MUST package (50), and preliminary phylogenies were reconstructed using FastTree v.2.1.7 (51). The examination of these preliminary trees revealed the presence in some of our data sets of highly divergent sequences, which generally clustered with their respective outgroup in the preliminary phylogenies and in some cases could be attributed to other functions based on searches carried out against the Pfam 28.0 (http://pfam.xfam.org/), PDB (http://www.rcsb.org/pdb/home/home.do), or KEGG (http://www.genome.jp/kegg/) database. These preliminary analyses were particularly important to determine the relationship of SC-TrpEG, SC-AroG, and SC-TrpD with their respective HK copies or with more distant relatives. We removed the sequences that were too divergent from our data sets, as well as those too short, too long, identical to others, or incomplete, as they could introduce unnecessary noise to our analyses. New MUSCLE v3.8.31 alignments were carried out on the curated data sets, and the statistical support of each alignment was checked using the Guidance server (http://guidance.tau.ac.il/ver2/) (52). The final alignments were trimmed using the program BMGE (53) and analyzed for similarity by neighbor-joining methods (total character distance) with nonparametric bootstrap analyses using PAUP* (54).

### Divergence time estimation methods.

Times to the most recent common ancestor (tmrca) were estimated using BEAST v1.8.1 (55). Four independent chains were run for each data set, and each was run for 50 million iterations and sampled every 5,000 iterations, initially. Some data sets were run for 100 million iterations to ensure convergence, and if the chain failed to converge, up to 8 additional runs were used to search for convergence. The posterior probability distributions were inspected manually using Tracer v1.6 (56). At least the first 10% of the samples were discarded as “burn-in,” and the 95% highest-posterior-density region (HPD) was calculated using Tracer. Phylogenies were summarized by TreeAnnotator v1.8.1 from the BEAST package. The tmrca were estimated using the following parameter settings: each data set was fitted with the LG amino acid substitution model (57) + Γ + invariant sites, and a Yule speciation model with uncorrelated lognormal-distributed relaxed clock model was employed. Random starting phylogenies were used under conditions in which the initial maximum age for a monophyletic cyanobacterium was set to 3,000 million (Ma) years ago. For time calibration, we used the appearance of filamentous cyanobacteria, and the appearance of heterocystous cyanobacteria as described in Results. The prior distribution for filamentous cyanobacteria was a truncated normal distribution with the mean 2,700 Ma and standard deviation 295, truncated between 2,700 and 3,200 Ma. The prior distribution for heterocystous cyanobacteria was set as an exponential distribution with mean of 210 Ma and offset (lower bound) of 2,100 Ma. The prior distribution for the age of the root node was a uniform distribution between 2,100 and 4,500 Ma. This means that the deepest node was constrained at the age of the Earth. All other priors used the default BEAST priors.
