# Supplementary material for: Timing the Evolutionary Advent of Cyanobacteria and the Later Great Oxidation Event Using Gene Phylogenies of a Sunscreen
Source: mBio. 2019 May 21;10(3):e00561-19. doi: 10.1128/mBio.00561-19 (PMC6529634; doi:10.1128/mBio.00561-19)

SUPPLEMENTARY FIGURE 1

Phylogenetic relationships of cyanobacteria derived from neighbor-joining analysis of amino acid sequences of the core scytonemin genes *scyABCF*. Numbers next to nodes represent non-parametric bootstrap support values based on 1000 replicates. Scytonemin-operon homologue sequences are demarcated by the orange bar, most closely related genes from other bacteria by the blue bar.

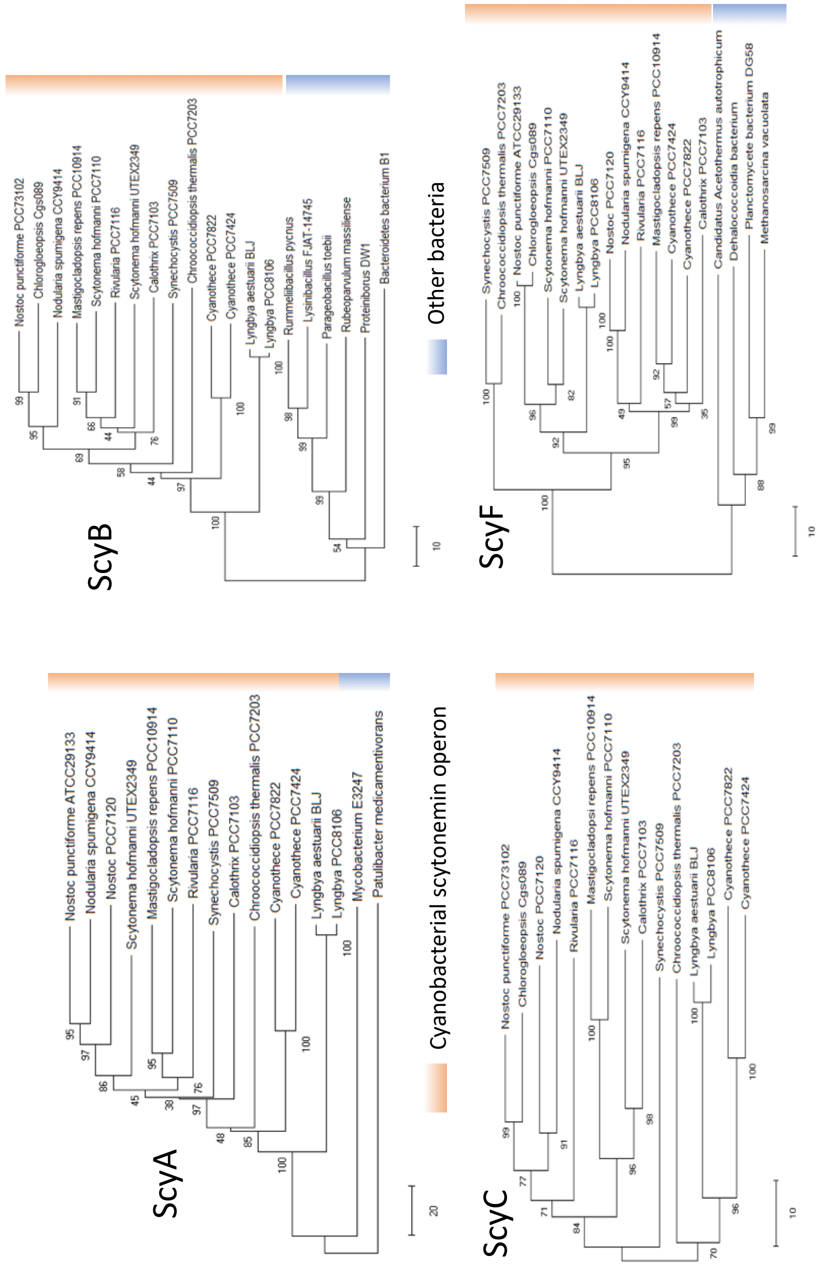

Supplement: FIG S1 [file mBio.00561-19-sf001.pdf]
