# Supplementary material for: Timing the Evolutionary Advent of Cyanobacteria and the Later Great Oxidation Event Using Gene Phylogenies of a Sunscreen
Source: mBio. 2019 May 21;10(3):e00561-19. doi: 10.1128/mBio.00561-19 (PMC6529634; doi:10.1128/mBio.00561-19)

SUPPLEMENTARY FIGURE 2

Phylogenetic relationships of cyanobacteria derived from neighbor-joining analysis of amino acid sequences of the *aroB* (114 taxa and 219 characters) and *aroG* (78 taxa and 341 characters) genes. Numbers next to selected nodes represent non-parametric bootstrap support values based on 500 replicates but only those above 70% are marked. Scytonemin-operon gene homologue sequences are shown in blue type, supernumerary homologues (beyond housekeeping and scytonemin-associated) and those in remnant scytonemin operons are in green type.

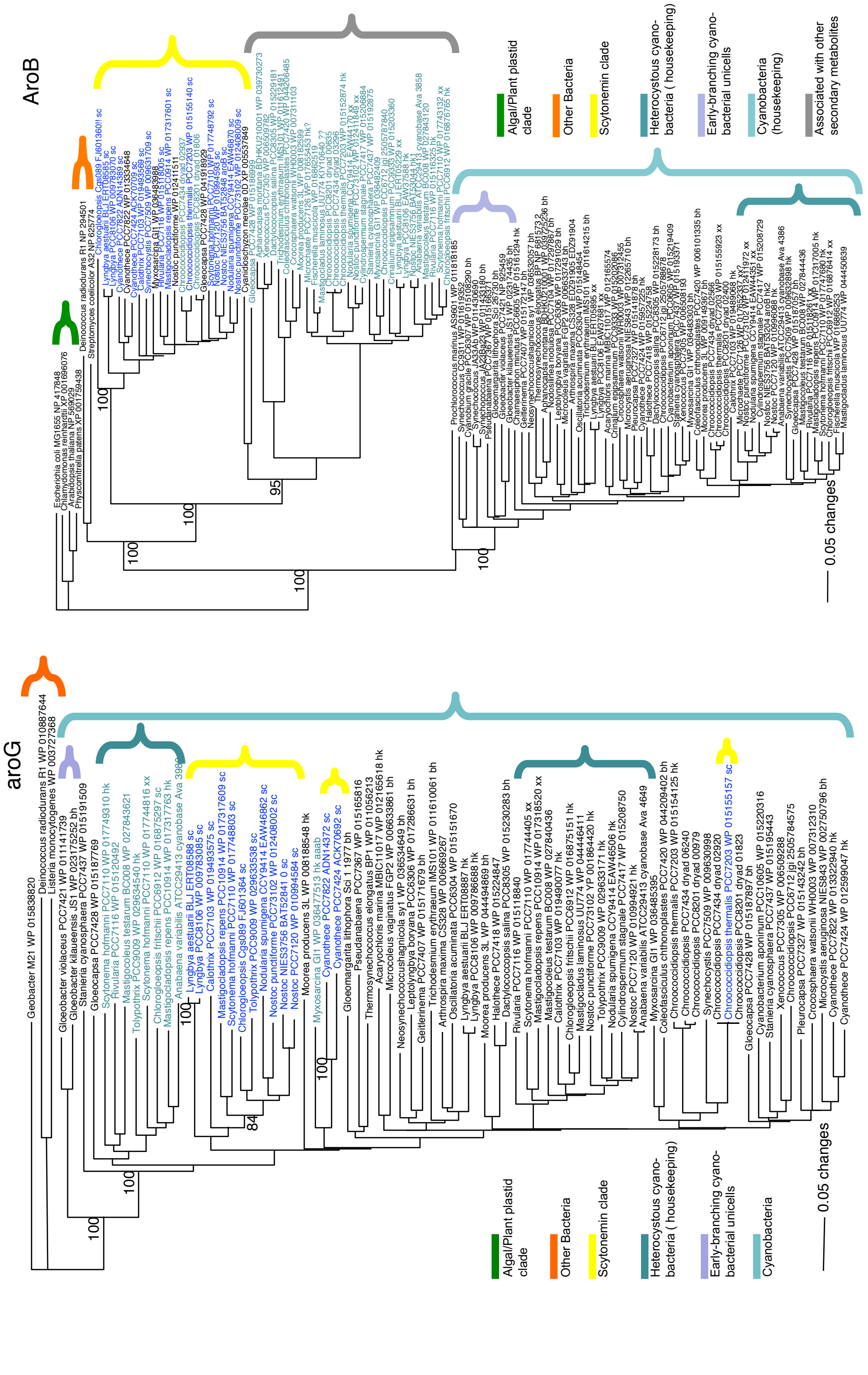

Supplement: FIG S2 [file mBio.00561-19-sf002.pdf]
