# Supplementary material for: Timing the Evolutionary Advent of Cyanobacteria and the Later Great Oxidation Event Using Gene Phylogenies of a Sunscreen
Source: mBio. 2019 May 21;10(3):e00561-19. doi: 10.1128/mBio.00561-19 (PMC6529634; doi:10.1128/mBio.00561-19)

SUPPLEMENTARY FIGURE 3

Phylogenetic relationships of cyanobacteria derived from neighbor-joining analysis of amino acid sequences of the *trpA* (89 taxa, 235 characters), *trpB* (89 taxa and 395 chracters), *trpC* (95 taxa and 233 characters), and *trpD* (84 taxa and 294 characters) genes. Numbers next to selected nodes represent non-parametric bootstrap support values based on 500 replicates but only those above 70% are marked. Scytonemin-operon gene homologue sequences are shown in blue type, supernumerary homologues (beyond housekeeping and scytonemin-associated) and those in remnant scytonemin operons are in green type.

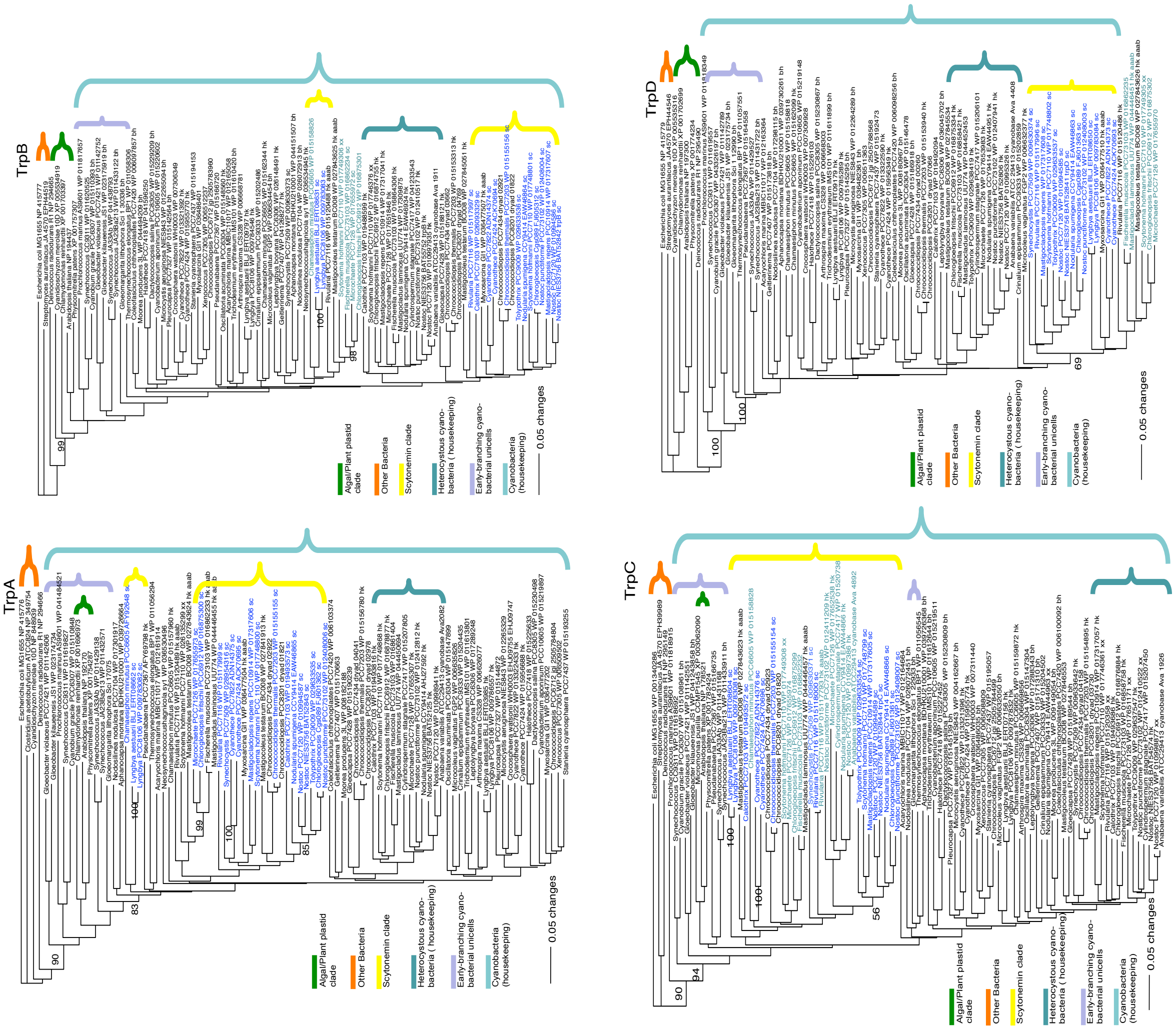

Supplement: FIG S3 [file mBio.00561-19-sf003.pdf]
