# Supplementary material for: Timing the Evolutionary Advent of Cyanobacteria and the Later Great Oxidation Event Using Gene Phylogenies of a Sunscreen
Source: mBio. 2019 May 21;10(3):e00561-19. doi: 10.1128/mBio.00561-19 (PMC6529634; doi:10.1128/mBio.00561-19)

SUPPLEMENTARY FIGURE 5

Phylogeny for scytonemin-dedicated AAAB genes of AroB amino acid sequences derived from BEAST analyses. Entries in blue type correspond to homologs found within full scytonemin operons. Bayesian posterior probabilities (BPP) at the nodes are color coded as follows: red for BPP ≥ 0.8, pink for 0.8 ≥ BPP ≥ 0.5 and white for BPP ≤ 0.5.

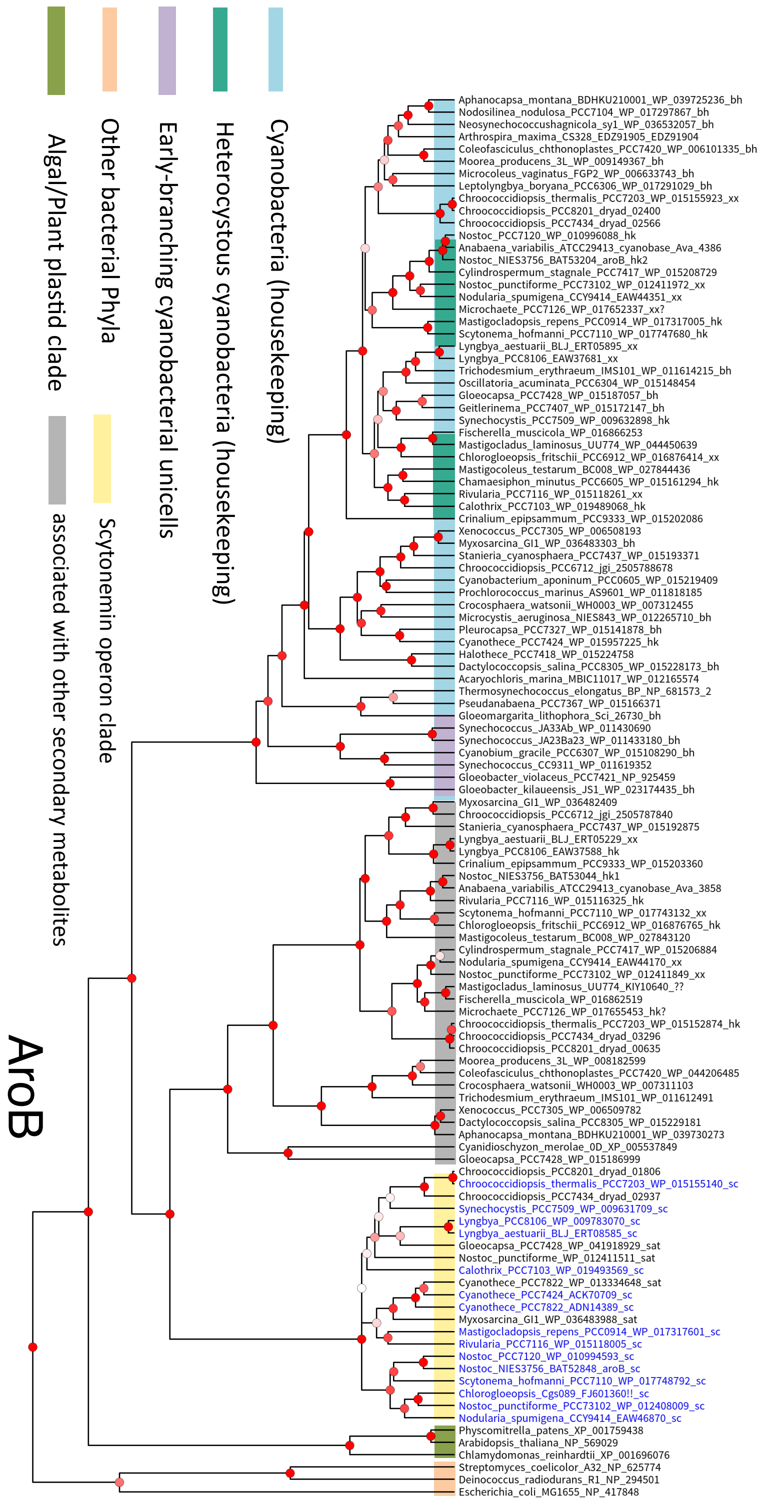

Supplement: FIG S5 [file mBio.00561-19-sf005.pdf]
