# Supplementary material for: Timing the Evolutionary Advent of Cyanobacteria and the Later Great Oxidation Event Using Gene Phylogenies of a Sunscreen
Source: mBio. 2019 May 21;10(3):e00561-19. doi: 10.1128/mBio.00561-19 (PMC6529634; doi:10.1128/mBio.00561-19)

SUPPLEMENTARY FIGURE 6

Phylogeny derived from BEAST analyses of AroG amino acid sequences for scytonemin-dedicated AAAB genes. Entries in blue type correspond to homologs found within full scytonemin operons. Those marked by a blue arrow, were found in remnant scytonemin operons or correspond to supernumerary homologues. Bayesian posterior probabilities (BPP) at the nodes are color coded as follows: red for BPP ≥ 0.8, pink for 0.8 ≥ BPP ≥ 0.5 and white for BPP ≤ 0.5. Red arrow marks the node corresponding to the most recent common ancestor of the oldest clade containing scytonemin-associated homologues.

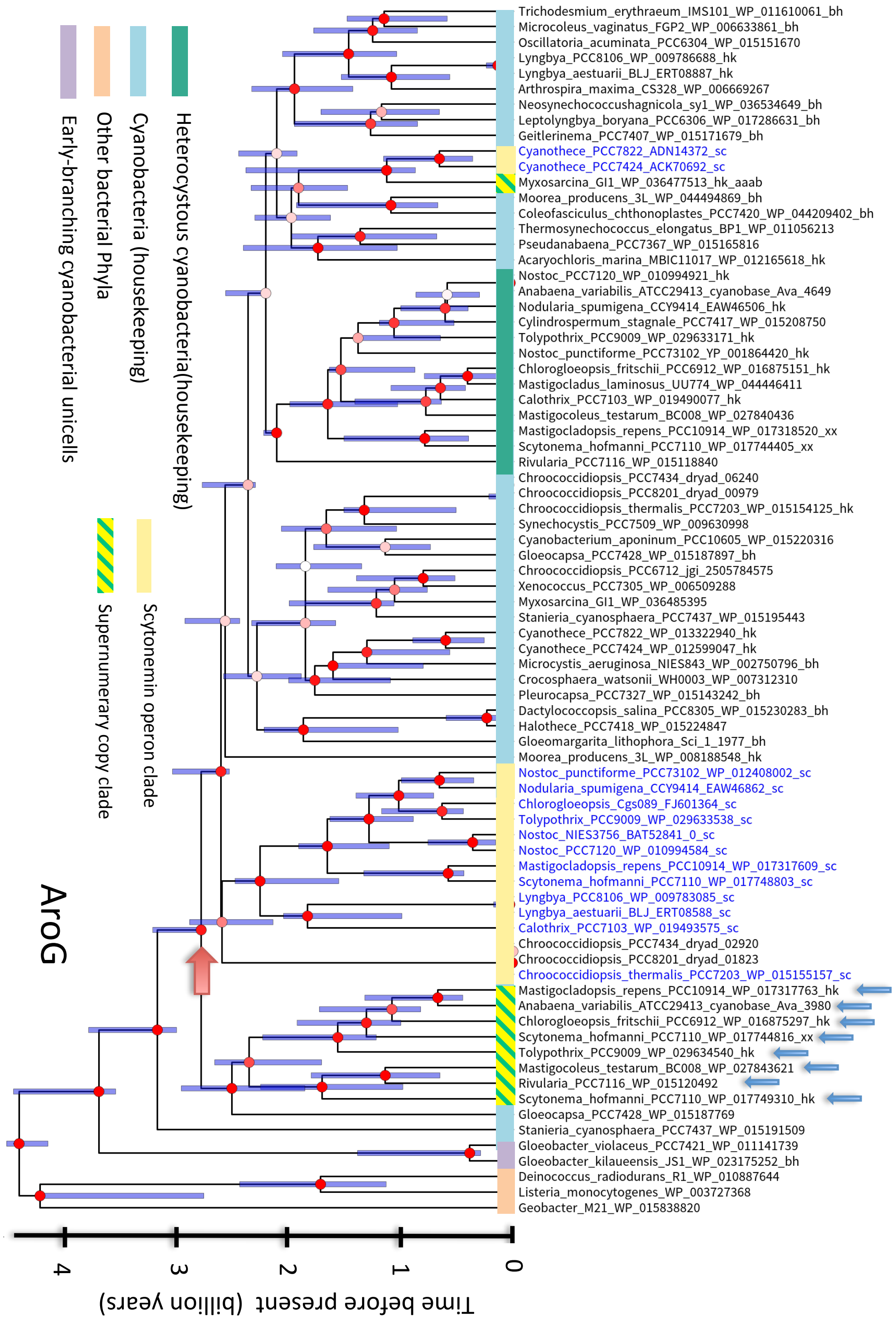

Supplement: FIG S6 [file mBio.00561-19-sf006.pdf]
