# Supplementary material for: Timing the Evolutionary Advent of Cyanobacteria and the Later Great Oxidation Event Using Gene Phylogenies of a Sunscreen
Source: mBio. 2019 May 21;10(3):e00561-19. doi: 10.1128/mBio.00561-19 (PMC6529634; doi:10.1128/mBio.00561-19)
Supplement: TEXT S1 [file mBio.00561-19-s0001.pdf]

SUPPPLEMENTARY FILE 10

A: Genomes used in the analyses.

Preliminary analyses

Cyanobacteria

Acaryochloris sp. CCMEE 5410
Acaryochloris marina MBIC11017
Anabaena cylindrica PCC 7122
Anabaena sp. 90
Anabaena variabilis ATCC 29413
Arthrospira maxima CS-328
Arthrospira platensis NIES-39
Arthrospira sp. PCC 8005
Calothrix desertica PCC 7102
Calothrix sp. PCC 6303
Chamaesiphon minutus PCC 6605
Chlorogloeopsis fritschii PCC 6912
Chroococcidiopsis sp. PCC 6712
Chroococcidiopsis thermalis PCC 7203
Coleofasciculus chthonoplastes PCC 7420
Crinalium epipsammum PCC 9333
Crocosphaera watsonii WH 0003
Cyanobacterium aponinum PCC 10605
Cyanobacterium sp. ESFC-1
Cyanobacterium stanieri PCC 7202
Cyanobium gracile PCC 6307
Cyanobium sp. PCC 7001
Cyanothece sp. ATCC 51142
Cyanothece sp. PCC 7425
Cylindrospermopsis raciborskii CS-505
Cylindrospermum stagnale PCC 7417
Dactylococcopsis salina PCC 8305
Fischerella muscicola PCC 7414
Fischerella sp. PCC 9605
Fischerella thermalis PCC 7521
Geitlerinema sp. PCC 7407
Geminocystis herdmanii PCC 6308
Gloeobacter kilaueensis JS1
Gloeobacter violaceus PCC 7421
Gloeocapsa sp. PCC 7428
Halothece sp. PCC 7418
Leptolyngbya boryana PCC 6306
Leptolyngbya sp. PCC 7376
Lyngbya aestuarii BL J
Lyngbya sp. PCC 8106
Mastigocladopsis repens PCC 10914
Mastigocoleus testarum BC008
Microchaete sp. PCC 7126
Microcoleus sp. PCC 7113
Microcoleus vaginatus PCC 9802
Microcystis aeruginosa NIES-843
Microcystis sp. T1-4
Moorea producens 3L
Nodosilinea nodulosa PCC 7104
Nodularia spumigena CCY9414
Nostoc punctiforme PCC 73102
Nostoc sp. PCC 7107
Nostoc sp. PCC 7120
Oscillatoria acuminata PCC 6304
Oscillatoria formosa PCC 6407
Oscillatoria nigro-viridis PCC 7112
Oscillatoria sp. PCC 7113
Planktothrix agardhii NIVA-CYA 126/8
Planktothrix prolifica NIVA-CYA 406
Planktothrix rubescens NIVA-CYA 407
Pleurocapsa sp. PCC 7327
Prochlorococcus marinus str. AS9601
Prochlorococcus marinus subsp. marinus str. CCMP1375
Prochlorococcus sp. W2
Prochlorothrix hollandica PCC 9006
Prochlorothrix sp. PCC 9006
Pseudanabaena sp. PCC 7367
Raphidiopsis brookii D9
Richelia intracellularis HM01
Rivularia sp. PCC 7116
Rubidibacter lacunae KORDI 51-2
Scytonema hofmanni PCC 7110
Spirulina major PCC 6313
Spirulina subsalsa PCC 9445
Stanieria cyanosphaera PCC 7437
Synechococcus elongatus PCC 6301
Synechococcus sp. CC9311
Synechococcus sp. JA-3-3Ab
Synechocystis sp. PCC 6803
Thermosynechococcus elongatus BP-1
Tolypothrix sp. PCC 9009
Trichodesmium erythraeum IMS101
Trichormus azollae ('Nostoc azollae' 0708)
Xenococcus sp. PCC 7305

Other bacteria

Pseudomonas aeruginosa PAO1
Caulobacter sp. K31
Deinococcus radiodurans R1
Clostridium acetobutylicum ATCC 824
Streptomyces aurantiacus JA 4570
Streptomyces coelicolor A32

Escherichia coli K 12
Geobacter sp. M21
Listeria monocytogenes

Eukaryotes (viridiplantae)
Cyanidioschyzon merolae
Galdieria sulphuraria
Chlamydomonas reinhardtii
Physcomitrella patens
Arabidopsis thaliana

Final analyses (cyanobacteria)

Chamaesiphon minutus PCC 6605	GenBank
Acaryochloris marina MBIC11017	GenBank
Anabaena variabilis ATCC 29413	CyanoBase
Aphanocapsa montana BDHKU210001	GenBank
Arthrospira maxima CS-328	GenBank
Calothrix PCC 7103	GenBank
Chlorogloeopsis fritschii PCC 6912	GenBank
Chroococcidiopsis sp. PCC 8201	Dryad
Chroococcidiopsis sp. PCC 6712	JGI
Chroococcidiopsis sp. PCC 7434	Dryad
Chroococcidiopsis thermalis PCC 7203	GenBank
Coleofasciculus chthonoplastes PCC 7420	GenBank
Crinalium epipsammum PCC 9333	GenBank
Crocosphaera watsonii WH 0003	GenBank
Cyanobacterium aponimum PCC 10605	GenBank
Cyanobium gracile PCC 6307	GenBank
Cyanothece PCC 7424	GenBank
Cyanothece PCC 7822	GenBank
Cylindrospermum stagnale PCC 7417	GenBank
Dactylococcopsis salina PCC 8305	GenBank
Fischerella muscicola PCC 73103	GenBank
Geitlerinema sp. PCC 7407	GenBank
Gloeobacter kilaueensis JS1	GenBank
Gloeobacter violaceus PCC 7421	GenBank
Gloeocapsa sp. PCC 7428	GenBank
Gloeomargarita lithophora	Moreira *et al*. (personal communication, now in GenBank)
Halothece sp. PCC 7418	GenBank
Leptolyngbya boryana PCC 6306	GenBank
Lyngbya aestuarii BL J	GenBank
Lyngbya sp. PCC 8106	GenBank
Mastigocladopsis repens PCC 10914	GenBank
Mastigocladus laminosus UU774	GenBank
Mastigocoleus testarum BC008	GenBank
Microchaete sp. PCC 7126	GenBank
Microcoleus vaginatus FGP-2	GenBank
Microcystis aeruginosa DIANCHI905	GenBank
Moorea producens 3L	GenBank
Myxosarcina sp. GI1	GenBank
Neosynechococcus sphagnicola sy1	GenBank
Nodosilinea nodulosa PCC 7104	GenBank
Nodularia spumigena CCY9414	GenBank
Nostoc punctiforme PCC 73102	GenBank
Nostoc sp. PCC 7120	GenBank
Nostoc sp. NIES-3756	GenBank
Oscillatoria acuminata PCC 6304	GenBank
Pleurocapsa sp. PCC 7327	GenBank
Prochlorococcus marinus AS 9601	GenBank
Pseudanabaena sp. PCC 7367	GenBank
Rivularia sp. PCC 7116	GenBank
Scytonema hofmanni PCC 7110	GenBank
Scytonema hofmanni UTEX 2349	GenBank
Stanieria cyanosphaera PCC 7437	GenBank
Synechococcus sp. CC9311	GenBank
Synechococcus sp. JA-2-3B	GenBank
Synechococcus sp. JA-3-3Ab	GenBank
Synechocystis PCC 7509	GenBank
Thermosynechococcus elongatus BP-1	GenBank
Trichodesmium erythraeum 21-75	GenBank
Xenococcus sp. PCC 7305	GenBank

B: Sequences from Nostoc punctiforme PCC 73102 (ATCC 29133) used as seeds in the phylogenetic analyses.

GenBank accesion # changes as work was being performed, so the headers contain both the older gi references from the original seeds and the current updated accession numbers (17-20-04).

AroG-sc

>gi|186681728|ref|WP_012408002.1| 3-deoxy-7-phosphoheptulonate synthase [Nostoc punctiforme PCC 73102]
MIIILKNGTPAEEITRISQELSETWKVTVEKSIGTHKVVLGLIGDTTSIDKLQIQEYSPWIEQVLR
VQQPFKRVSREFRHGEASEVVVPTPNGDVYFGEHHPIVVVAGPCSVENEAMIVETAKRVKAA

GAKFLRGGAYKPRTSPYAFQGYGESALDLLAAAREATGLGIVTELMDAADLSAVARTADIIQI
GARNMHNFSLLKKVGAQDKPVLLKRGMSATIDEWLMAAEYILASGNPNVILCERGIRTFDGK
YARNTLDLSVLPVLRSLTHLPIMIDPSHGTGRSEYVPSMAIAAIAAGTDSLMIEVHPNPAKALS
DGPQSLTPDKFDRLVQEMSIIGKVVDRWSTPTFDSINDNRILFAPELSK

AroG

>gi|186463676|gb|ACC79477.1| phospho-2-dehydro-3-deoxyheptonate aldolase [Nostoc punctiforme PCC 73102]

MIIVMKTGSPEAEINRIDEELTSWGLTPEKIVGQHKVVIGLVGETASLDPLQIQELSPWIEQVLR
VEQPYKRASRQYRHGEASEVVVNTPNGAVVFGEHQPLVVVAGPCSVENEEMIVETARRVKT
AGAKFLRGGAYKPRTSPYAFQGHGESALDLLARAREVSGLGIITEVMDAADLDKIVEVADVIQ
VGARNMQNFSLLKKVGAQPKPVLLKRGMAATIEDWLMAAEYVLASGNSNVILCERGIRTFDR
QYTRNTLDLSVVPVLRKLTHLPIMIDPSHGVGWSEFVPSMAMAAIAAGTDSLMIEVHPNPAKA
LSDGPQSLTPDRFDNLMQELAVIGKAVGRWQQPAVALA

AroB-sc

>gi|186681735|ref|WP_012408009.1| 3-dehydroquinate synthase [Nostoc punctiforme PCC 73102]

MIVDIKQKSRLIHQRVSVSFNYEVYFTQNLFELKNPTLAQVISADEETKPKKVVAVIDAGILKY
QPELVKQLVAYTKFYGEVLAIAAEPMIISGGEAAKNDRTLVEQIQQLIETAGLCRHSYVLAIGG
GAVLDLVGYAAATAHRGIRLIRVPTTVLAQNDSGVGVKNGINAFGKKNFLGTFAPPYAVINDS
AFLTTLDDRDWRSGIAEAVKVALIKDANFFDFIHSHSKALGRRDMDAMQQVIYRCAQLHLEHI
ANSGDPFEMGSSRPLDFGHWAAHKLEHLTNYRLRHGEAVAIGIALDSTYSYLAGLLDCSEWQ
RILNTLSALGFTLYVPELAQKLSQQEHPDCLFRGLTEFREHLGGELTLTLLKNIGKAIEVHEVNL
LLYRQAISLLQEF

AroB

>gi|186685776|ref|WP_012411972.1| 3-dehydroquinate synthase [Nostoc punctiforme PCC 73102]

MTSLINVNLPEQSYEIAIAPSNLDQLGQQMANLKLGKKVLLVSNPTIFKHYGERAITSLKSAGF
EVASCTLPPGERYKNLNSIQKLYDVALENRLERSATMVALGGGVIGDMTGFAAATWLRGINV
VQVPTTLLAMVDSAIGGKTGVNHPHGKNLIGAFHQPRLVLIDPDVLKTLPMREFRAGMAEVIK
YGVIWDAELFAQLEASKRLDQLRYVKPELIDSILTRSCQAKADVVGKDEKEGGLRAILNYGHT
IGHAVESLTGYRLVNHGEAVAIGMVAAGQIAVELGMWQKEDTERQNALIQKTGLPTQLPSGV
DIEAIIDALQLDKKVKAGKVRFVLPTEIGVVTVTDEVPSDIIRQVLQGM

TyrA-sc

>gi|186681737|ref|WP_012408011.1| prephenate dehydrogenase [Nostoc punctiforme PCC 73102]

MPLKSSYSDQLKKTDQSLIALLSDRISLLASEQPSLDEQLASVAPLLAQAGIPESVWAGVVNSC
HASLTPKSAINHASPRQVTIIGGRGRMGRLFQEQLSLVGHNVSILEHEDWEYAEQLLSQAELVL
VSVPIEHTVDVIKRAAKYLASNTALCDITSIKTQPTQAMLEHHCGPVMGLHPMFGPNIKSFLGQ
KVVVCPGRNDDSFQWLLDFLKSKGGELIVCTPEEHDRMMVIIQATQHFCRFSLGVFLAQARVE

IEQSLTMSTPNYRQEIDIVKRLFAQNPNLCVDIMLATEERCNAISFLANTYSRLARLVARKDRE
ALIKEFENTQSFFEGKINSFLQPLNTTALKRDFKPQNIGI

TyrA

>gi|186681456|ref|WP_012407731.1| arogenate dehydrogenase [Nostoc punctiforme PCC 73102]

MSKLKMNIGILGLGLIGGSLGFDLRSQGHHILGVSRRESTCQKAVAIGSVDEASVDLSLLAAAE
VVFICTPLALIVPQLEQMIAHLSTATIVTDVGSAKAQIVKAISPLWDNFIGGHPMAGRTDSGIEA
AQRNLFVDKPYVLTPIATTPTSAIAVVEEIVRSLGANIYYCQPEQHDRAVSWISHLPVMVSSSLI
AACLSETDPDVLELAQKLASSGFRDTSRVGGGNPELGVMMARYNRQALLRSLQQYRHNLDE
LTNLIEQENWTVLEQKLKSTGKARPDFVD

TrpD-sc

>gi|186681729|ref|WP_012408003.1| anthranilate phosphoribosyltransferase [Nostoc punctiforme PCC 73102]

MIAVTQTPPDNIAIPSEFYNWSALLQQLLNRQSLTVPQAADLMQGWLTDAIPHVLSGAILAAIQ
AKGVSAQELVGMASVLQSQSPIPSTQYPAPLIDTCGTGGDGASTFNISTAVAFVAAAAGVKVA
KHGNRSASSKTGSADVLEALGINLNATPEKVQAAVGEVGITFLFAPGWHPALKAIASLRKTLK
VRTVFNLLGPLVNPMRPTGQIIGVNDPLLLEEIVQALSQLGCQQAIAVHGRERLDEAGLADVT
DLAVLQDKKVRSLTLNPQELGLNFAPTAALCGGDVQENADILKAVLQGKGTQAQQDVVALN
TALAFQVGEAIHGETDVLASCVKGIAFAKEILQSGAAWTKLEQLAEFLR

TrpD

>gi|14594711|gb|AAK68645.1| anthranilate phosphoribosyltransferase [Nostoc punctiforme PCC 73102]

MTTFPISAQEASASWYMLLQQLIDGQSLSRTQAAELMQGWLSEAVPPELSGAILTALNFRGISA
DELTGMAEVLQSQSSPLSTQHGLNAPLSTALSTVIDTCGTGGDGSSTFNISTAVAFVAAASGVP
VAKHGNRSASSLTGSADVLEALGVNLSASSDKVQAALQEVGITFLFAPGWHPALKAVAQLRR
TLRVRTVFNLLGPLVNPLRPSGQVVGLFTPKLLATVAQALNNLGKEKAIVLHGREKLDEAGLG
DETDLAVLSDGEVQVTTINPLDLDLTPAPIGMLRGGDVQENAVILKAVLQGKGTQAQQDAVA
LNASLALQVAGTIALLDHAQGIKIAKDILQSGAAWTKLEQLVEFLGN

TrpC-sc

>gi|186681733|ref|WP_012408007.1| indole-3-glycerol-phosphate synthase [Nostoc punctiforme PCC 73102]

MTNQVTASRHILEEIVLHKRQEVAQMQQELPLASLQQQLITAPPVRNFLAALQQNPNQPSLIAE
VKKASPSRGIIRADFDPVAIAQAYERGGAACLSVLTDRKFFHGSFDNLRNVRSHVALPLLCKEF
IIDPYQIYLARTAGADAVLLIAAILSDQELQNFLQVIHDLGMNALVEVHTLAELDRVLKLDDLH
LVGINNRNLEDFTVDLGITQQLLAQRQQYLQSLDITVVSESGLYTPADLSLVAEAGVRAVLVG
ESLVKQSDVEQAVRSLLRLS

TrpC

>gi|186684302|ref|WP_012410522.1| indole-3-glycerol-phosphate synthase [Nostoc punctiforme PCC 73102]

MQIRRRSPNPAIAVSMLRYQAAVPDSAPNNILEEIVWQKEVEYDLMREKVPLQELRKQVLTAP
PTRDFVAALRQGKTKPALIAEVKKASPSKGVFREDFDPVAIAQSYQQGGASCLSVLTDVKFFQ
GSFENLAKIRAAVDLPLLCKEFIIYAYQIYLARIHGADAILLIAAILGDQDLKYFLKIANNLKMA
ALIEVHSLAELDRVLALDGVSLVGINNRNLEDFSVDLQTTCQLLAARGSQLQEKNILVVSESGL
HNPDNLSLVLTAGASAVLIGESLVKQPDPGAAIASILPKNF

TrpA-sc

>gi|186681732|ref|WP_012408006.1| tryptophan synthase subunit alpha [Nostoc punctiforme PCC 73102]

MTSISNSFQTLRDRQQCALIPFITAGDPNLETTAQALRILDRNGADFIELGIPYSDPLADGPVIQA
AATRALQKGTKLEQVLEMLQAVTPTLKAPIILFTYYNPILHRGIKTFLAQIAAVGVQGLVVPDL
PLEEAEELIQTAASFGIEVILLVAPTSSKDRILAIARQSQGFIYLVSVTGVTGIRAQIQDRVKDLIT
DLRSVTDKPIGVGFGISGPEQAHQVREWGADAVIVGSAFVKRLAEGSPTERLQAVEKLCQELK
AAITPVSLQQVVSAK

TrpA

>gi|186686628|ref|WP_012412812.1| tryptophan synthase subunit alpha [Nostoc punctiforme PCC 73102]

MTAISDCFETLGHNHECALIPFITAGDPDLETTAKALQVLDQSGADIIELGIPYSDPLADGPVIQ
AAATRALQRGTKLEHVLEMLQGITPKLRSPIVLFTYYNPILHRGIDKFLQEIAAAGVAGLVVPD
LPLEEAAGLLEPAKEMGIDVILLVAPTSDAKRIEAIAHSSQGFIYLVSVTGVTGVRSQLESRVSD
LLKQIRGVTEKPIGVGFGISDAAQARQVKEWGADAAIVGSAVVKRLAEGTPEQGLSAIAQLCQ
SLKAAIKTSTNTPLD

TrpB-sc

>gi|186681730|ref|WP_012408004.1| tryptophan synthase subunit beta [Nostoc punctiforme PCC 73102]

MVSIQDIKTATIQPDSLGRFGRFGGKYVPETLMPALSELEAAFQKYRNEPSFQAELQNLLRDYV
GRPSPLYFAERLTANYARPDGTGPQIYLKREDLNHTGAHKINNALAQVLLAKRMGKQRVIAE
TGAGQHGVATATVCARFGLRCVIYMGIHDMERQALNVFRMKLMGAEVHPVEAGTGTLKDA
TSEAIRDWVTNVETTHYILGSVAGPHPYPMIVRDFHAIIGIETRAQSQEKWEGLPDILLACVGG
GSNAIGLFYEFLNEPSIRLIGVEAAGEGVNTEKHAATLTKGKIGVLHGAMSYLLQDDDGQIIEA
HSISAGLDYPGVGPEHSYLKDLGRAEYYSVTDKQALEAFQRLSQLEGIIPALETAHAIAYLETL
CPELEGSPRIVINCSGRGDKDVQTVAKVLIP

TrpB

>gi|186684297|ref|WP_012410517.1| tryptophan synthase subunit beta [Nostoc punctiforme PCC 73102]

MTTTPLSPSSTAQVPDALGRFGRFGGKYVPETLMPALAELETAYQQYRNEPGFQAELQQLLRD
YVGRATPLYFAERLTAHYARPDRTGPQVYLKREDLNHTGAHKINNALGQVLLAKRMGKQRII
AETGAGQHGVATATVCARFGLECVIYMGVHDMERQSLNVFRMRLMGAEVRPVEAGTGTLK
DATSEAIRDWVTNVETTHYILGSVAGPHPYPMMVRDFHAVIGQETRTQAMEKWGTLPDILLA
CVGGGSNAMGLFYEFINEPSIRFIGIEAAGEGVNTEKHAATLTKGRVGVLHGAMSYLLQDEDG
QIIEAHSISAGLDYPGVGPEHSYLKDTGRAEYYSVTDAEAMAAFQRLSKLEGIIPALETAHAIA
YLETLCPQLSGSPRIVINCSGRGDKDVQTVAKLLDPA

TrpEG-sc

>gi|186681734|ref|WP_012408008.1| anthranilate synthase [Nostoc punctiforme PCC 73102]

MIFNSRSYKTLGGVIVSRSITEVKMDTALEDILFHLNSQRGGLLRSSYEYPGRYKRWAIGFVNP
PLELTTQENAFNLIALNERGLVLLPLLLECLSNSLQLEKVTLDNNNIVGFVKSTKRLFTEEERSK
QPSAFTVVREILHTFSSQEDEHLGLYGAFGYDLVFQFEPITQSLERPKDQRDLVLYLPDELIVVD
YYQQRAFRLQYDFETAHGNTKNLPRTGESVDYRGKHLTPNETADHKVGEYAKKVESALDYF
RRGDLFEVVPSQNFFEGYEDEPSKLFETLKDINPSPYGFIFNLGGEYLIGASPEMFVRVEGKRVE
TCPISGTISRGLDALDDAVQIRHLLNSHKDEAELTMCTDVDRNDKSRICEPGSVQVIGRRQIEL
YSHLIHTVDHVEGTLRSQFDALDAFLSHTWAVTVTGAPKKAAIDFIEQHERSARRWYGGAVG
YLNFNGNLNTGLILRTIRLKDCIAEVRVGATVLYDSIPQAEEQETITKAAALFETIRRVKQSSHK
IDESSSIKSTKILPCVATGKRILLIDYEDSFVHTLANYIRQTGASVTTLRHGFSETLFDTERPDLV
VLSPGPGRPSDFRVPQTVGALLHRKIPIFGVCLGLQSIVEAFDGQLGVLNYPQHGKSSRIFVTDS
DSVTFKNLPESFTVGRYHSLFALPQHLPQELKVTAISDDNVIMGIEHQTLPIAAVQFHPESIMTL
AGEVGLEIIKNVVRAYTQVEESLVIGH

TrpE

>gi|186685226|ref|WP_012411432.1| anthranilate synthase component I [Nostoc punctiforme PCC 73102]

MIFPHFSQFSSLAQQGNFVPVYQEWVADLDTPVSAWYKVCAGQPYSFLLESIEGGEKLGRYSL
VGCDPLWVLEARGDRTTQLNRDGSQVVFAGDPFAALAECLAPYHPVKLPELPPGIGGLFGFW
GYELINWIEPRVPIYPPDERNIPDGLWMQVDHLLIFDQVKRKIWAIAYADLRDPNVDLHAAYQ
QACDRVTQMLEKLSLPLSPEKTRLEWKSPGSRGAEEQKSGETDEYNSNFTRPDFCASVQKAK
DYIKAGDIFQVVISQRLSTPYTGDPFALYRSLRQINPSPYMAYFNFQDWQIIGSSPEVMVKAET
APDGGVIATVRPIAGTRPRGKTTQQDAAFAQDLLEDPKEVAEHVMLVDLGRNDLGRVCQNGS
VKVDELMVVERYSHVMHIVSNVVGKLALDKTAWDLLKACFPAGTVSGAPKIRAMEIIHELES
SRRGVYSGVYGYYDFEGQLNTAIAIRTMVVHDKTVSVQAGAGLVADSEPEKEYEETLNKARG
LLEAIRCLR

TrpG

>gi|186681805|ref|WP_012408079.1| glutamine amidotransferase [Nostoc punctiforme PCC 73102]

MIIVIDNYDSFTYNLVQYLGELAAEFSVADDIKVFRNDKISIDEIRALKPEAVVISPGPGRPEDA
GISLELIEQLGQELPILGVCLGHQSIGQVFGGKIIPAPELMHGKTSQVSHTGVGVFRGLENPLIAT
RYHSLVIERETCPDVLEITAWVEDNTIMGVRHRNYPHIQGVQFHPESVLTSSGKQLLRNFLEQL
QSR
